# Complete genome sequences of α-glucosidase-positive and -negative strains of *Nocardia seriolae* from *Seriola* species in Japan

**DOI:** 10.1128/MRA.00712-23

**Published:** 2023-09-26

**Authors:** Kousuke Umeda, Yuta Matsuura, Yoshiko Shimahara, Tomokazu Takano, Tomomasa Matsuyama

**Affiliations:** 1 Nansei Field Station, Fisheries Technology Institute, Japan Fisheries Research and Education Agency, Mie, Japan; 2 Kamiura Field Station, Fisheries Technology Institute, Japan Fisheries Research and Education Agency, Oita, Japan; Rochester Institute of Technology, Rochester, New York, USA

**Keywords:** *Nocardia seriolae*, *Seriola dumerili*, *Seriola quinqueradiata*, α-glucosidase, hybrid assembly

## Abstract

We report complete genome sequences of two strains of *Nocardia seriolae*, the causative agent of nocardiosis in fish. Strains KGN1266 (α-glucosidase-positive) and 024013 (α-glucosidase-negative) were isolated from *Seriola dumerili* and *Seriola quinqueradiata*, respectively. Whole genome sequences were hybrid-assembled using Oxford Nanopore long-read and BGI DNBseq short-read sequencing.

## ANNOUNCEMENT

*Nocardia seriolae* is the causative agent of nocardiosis in fish. Previous studies reported that strains of *N. seriolae* are divided into two groups by α-glucosidase activity and that α-glucosidase-negative strains were more prevalent in Japan (1, 2). There are some complete genomes deposited in the public databases, but none of them are from α-glucosidase-negative strains. Here, we determined the complete genome sequence of two new strains, α-glucosidase-positive KGN1266 and α-glucosidase-negative 024013, using a hybrid approach combining long-read and short-read sequencing.

The strains were originally isolated from diseased fish in fish farms and stored at −80°C until use. Detailed strain information is shown in Table 1. Bacterial cells were cultivated at 25°C for 5 d in brain heart infusion broth (Becton, Dickinson and Company, Franklin Lakes, NJ, USA). Pelleted cells were pretreated with lysozyme (20 mg/mL) and 1% Triton X-100 in TE buffer (pH 8.0) for 30 min at 37°C. Genomic DNA was extracted using the Maxwell 16 Tissue DNA Purification Kit (Promega Corporation, Madison, WI, USA). DNA concentration and quality were assessed using a BioSpec-nano (Shimadzu Corporation, Kyoto, Japan). All the kits used for DNA extraction and library preparation were used by following the manufacturer’s instructions throughout the study, unless otherwise stated.

**TABLE 1:** Strain information and statistics of sequencing, assembly, and annotation of the reported *N. seriolae* strains

Parameter		Data for strain:	
		KGN1266	024013
Strain information	Collection date	5 Nov. 2012	24 Oct. 2002
	Geographical origin	Kagoshima, Japan	Oita, Japan
	Host species	*Seriola dumerili*	*Seriola quinqueradiata*
	Isolation source	Brain	Muscle
	α-glucosidase activity	Positive	Negative
Long read	Sequencing technology	Nanopore GridION	Nanopore MinION
	No. of raw reads	90,144	296,925
	N50 of raw reads (bp)	15,432	3,190
	No. of filtered reads	88,316	158,719
	Total size of raw reads (bp)	1,160,497,591	547,607,173
	Total size of filtered reads (bp)	1,159,496,130	477,070,115
	Coverage	141.0	58.8
Short read	Sequencing technology	BGI DNBseq	BGI DNBseq
	Read length (bp)	2 × 200	2 × 150
	No. of raw read pairs	16,054,624	4,108,358
	No. of filtered read pairs	12,055,655	3,780,456
	Total size of raw reads (bp)	6,421,849,600	1,232,507,400
	Total size of filtered reads (bp)	4,822,262,000	1,134,136,800
	Coverage	586.5	139.8
Assembly statistics	Genome size (bp)	8,222,513	8,113,213
	GC content (%)	68.1	68.1
Annotation data	No. of protein coding sequences	8,045	7,815
	No. of rRNAs	12	12
	No. of tRNAs	74	72

For long-read sequencing, DNA libraries were prepared using the ligation sequencing kit SQK-RAD004 (Oxford Nanopore Technologies [ONT], Oxford, UK) without shearing. The libraries were sequenced on a GridION or MinION system with a Flongle flow cell (R9.4.1; ONT). Generated fast5 files were basecalled with Guppy v.6.4.6 in the high accuracy model. Reads ≥1,000 bp were selected using Filtlong v.0.2.1 (3).

Short-read sequencing was performed on DNBSEQ-G400 platform (BGI Genomics, Shenzhen, China). For strain KGN1266, genomic DNA was sheared to average 400 bp fragments with an S2 device (Covaris, Woburn, MA, USA). The DNA library was then prepared using MGIEasy Universal DNA Library Prep Set (MGI Tech, Shenzhen, China). For strain 024013, the DNA library was prepared using BGI Optimal DNA Library Prep Kit (BGI Genomics). The raw reads were adapter-trimmed and filtered with a quality threshold of 30 using SOAPnuke v.2.1.7 (4).

The genome of strain KGN1266 was assembled from the long reads using Flye v.2.9.2 (5). The Flye assembly was manually curated based on the sequencing depth of each contig using Bandage v0.8.1 (6). The genome of strain 024013 was assembled from the long reads and short reads using Unicycler v.0.5.0 (7). Both assembled genomes were finalized by three-round polishing with Pilon v.1.24 (8) using Minimap2 long-read (9) and BWA-MEM2 short-read (10) alignments. The complete genomes were annotated using DFAST v1.2.18 (11) with settings of “—fix_origin” and “—offset 100.” Default parameters were used for all software except where otherwise noted.

**Fig 1:**
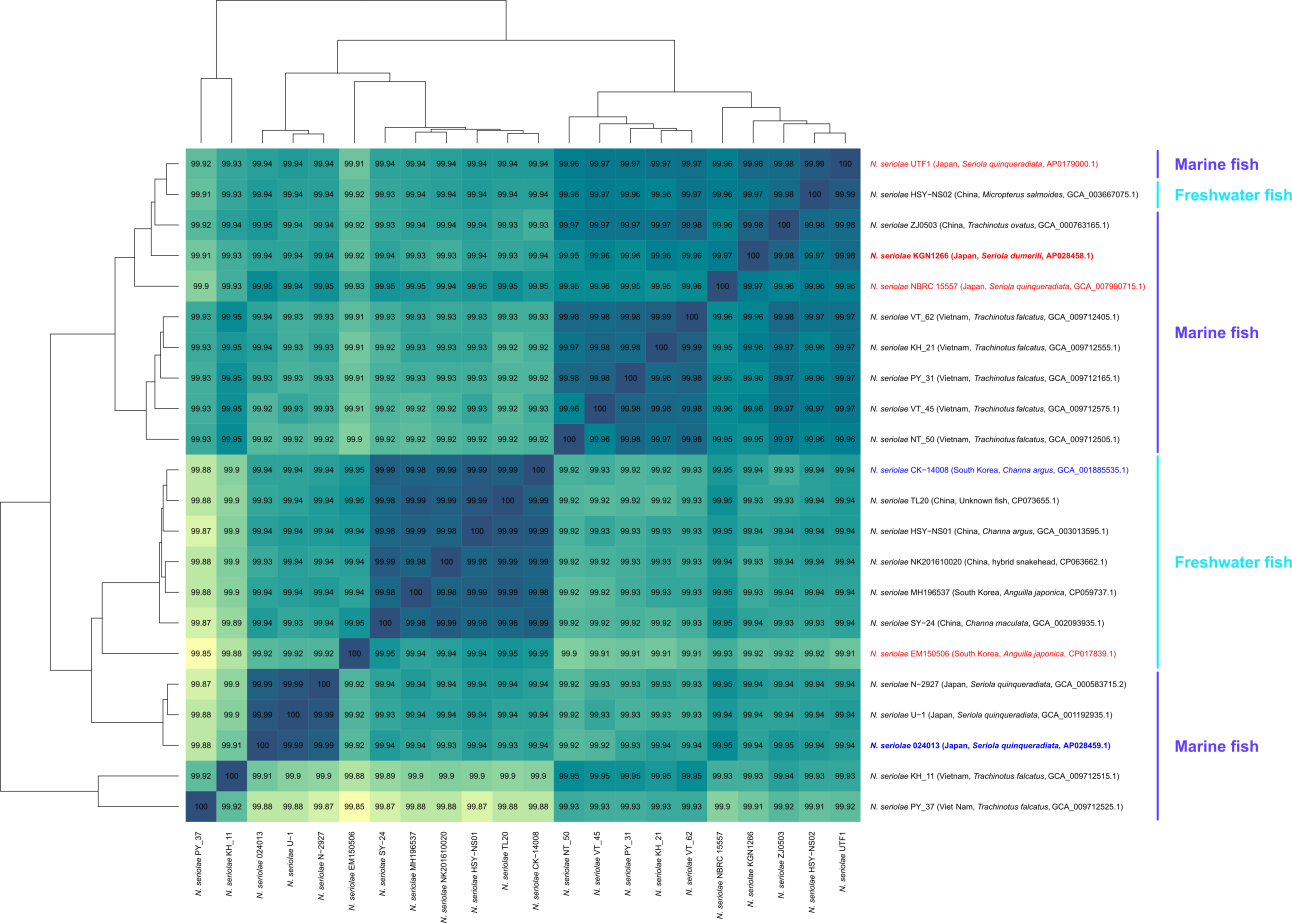
Average nucleotide identity percentage matrix for *N. seriolae* genome sequences. The sequences were determined in this study or were obtained from public databases. The heatmap and dendrogram were generated using the heatmap.2 function in the R package gplots (13). Geographical origins, host species, and GenBank accession numbers are shown in parentheses. Red, blue, and black characters represent positive, negative, and unknown α-glucosidase activity, respectively. Bold characters represent the sequences determined in this study.

Genome assembly statistics and annotation features are presented in Table 1. Average nucleotide identity (ANI) values were calculated using the ANI Calculator (OrthoANIu v1.2) (12) (Fig. 1). *N. seriolae* strains were roughly divided into groups of freshwater and marine fish. The data will help us understand the mechanisms of pathogenicity and evolutionary relationships among strains of *N. seriolae*.

## Data Availability

The complete genome sequences have been deposited in DDBJ/GenBank under accession nos. AP028458 (strain KGN1266) and AP028459 (strain 024013). The versions described in this paper are AP028458.1 and AP028459.1. The raw sequence reads have been deposited in DDBJ Sequence Read Archive (DRA) under accession nos. DRR491488, DRR491489, DRR491490, and DRR491491, BioSample accession nos. SAMD00623494 and SAMD00623495, and BioProject accession no. PRJDB15982.
